# Human genetic determinants of the gut microbiome and their associations with health and disease: a phenome-wide association study

**DOI:** 10.1038/s41598-020-70724-5

**Published:** 2020-09-08

**Authors:** Hilde E. Groot, Yordi J. van de Vegte, Niek Verweij, Erik Lipsic, Jacco C. Karper, Pim van der Harst

**Affiliations:** 1 Department of Cardiology, University Medical Center Groningen, University of Groningen, Hanzeplein 1, 9700 RB Groningen, The Netherlands; 2 Department of Cardiology, Division Heart and Lungs, University Medical Center Utrecht, University of Utrecht, Utrecht, The Netherlands

**Keywords:** Risk factors, Cardiology, Cardiovascular biology, Translational research

## Abstract

Small-scale studies have suggested a link between the human gut microbiome and highly prevalent diseases. However, the extent to which the human gut microbiome can be considered a determinant of disease and healthy aging remains unknown. We aimed to determine the spectrum of diseases that are linked to the human gut microbiome through the utilization of its genetic determinants as a proxy for its composition. 180 single nucleotide polymorphisms (SNPs) known to influence the human gut microbiome were used to assess the association with health and disease outcomes in 422,417 UK Biobank participants. Potential causal estimates were obtained using a Mendelian randomization (MR) approach. From the total sample analysed (mean age was 57 ± 8 years), 194,567 (46%) subjects were male. Median exposure was 66-person years (interquartile range 59–72). Eleven SNPs were significantly associated with 28 outcomes (Bonferroni corrected *P* value < 4.63·10^−6^) including food intake, hypertension, atopy, COPD, BMI, and lipids. Multiple SNP MR pointed to a possible causal link between *Ruminococcus flavefaciens* and hypertension, and *Clostridium* and platelet count. Microbiota and their metabolites might be of importance in the interplay between overlapping pathophysiological processes, although challenges remain in establishing causal relationships.

## Introduction

It has been suggested that different diseases may share more pathophysiological mechanisms than initially assumed^1–6^. An enhanced understanding of these complex shared disease causing mechanisms is of paramount importance to further improve our strategies to study, prevent and treat diseases.

One of the possible shared systems is the human gut microbiome, which has been suggested as a key inter-player in a variety of individual disease entities^7^. For example, the gut microbiome has been linked to immune system activation, inflammatory processes and metabolic phenotypes. Moreover, it has been associated with thrombosis and the development of cardiovascular disease (CVD)^8–17^, Alzheimer's disease, and cancer^18–20^. Human genetic variants have been linked to the microbiome composition^21–23^ and with this knowledge, rather than actual measurements of the microbiome itself, human genetic variants can be used as a proxy to inform about human gut microbiome composition. This is of interest because large cohorts in which genetic information is available can be studied even if the microbiome itself has not been measured. In addition, the use of genetic determinants can be useful to study the potential long-term health effects of having increased or decreased levels of specific gut microbiota.

Although several associations between the human gut microbiome and individual diseases have been reported, a broad characterization of a large spectrum of health and disease states is lacking but is highly desired as it may help identify potentially common mechanistic pathways^17,24,25^. Hence, this study aimed to explore the associations between known genetic determinants of the human gut microbiome and the presence of health and its determinants (absence of disease, vital and blood biomarkers and food intake) and disease states in a very large and comprehensive population cohort and investigate possible causal mechanisms using a Mendelian randomization (MR) approach.

## Results

### Population characteristics

**Figure 1:**
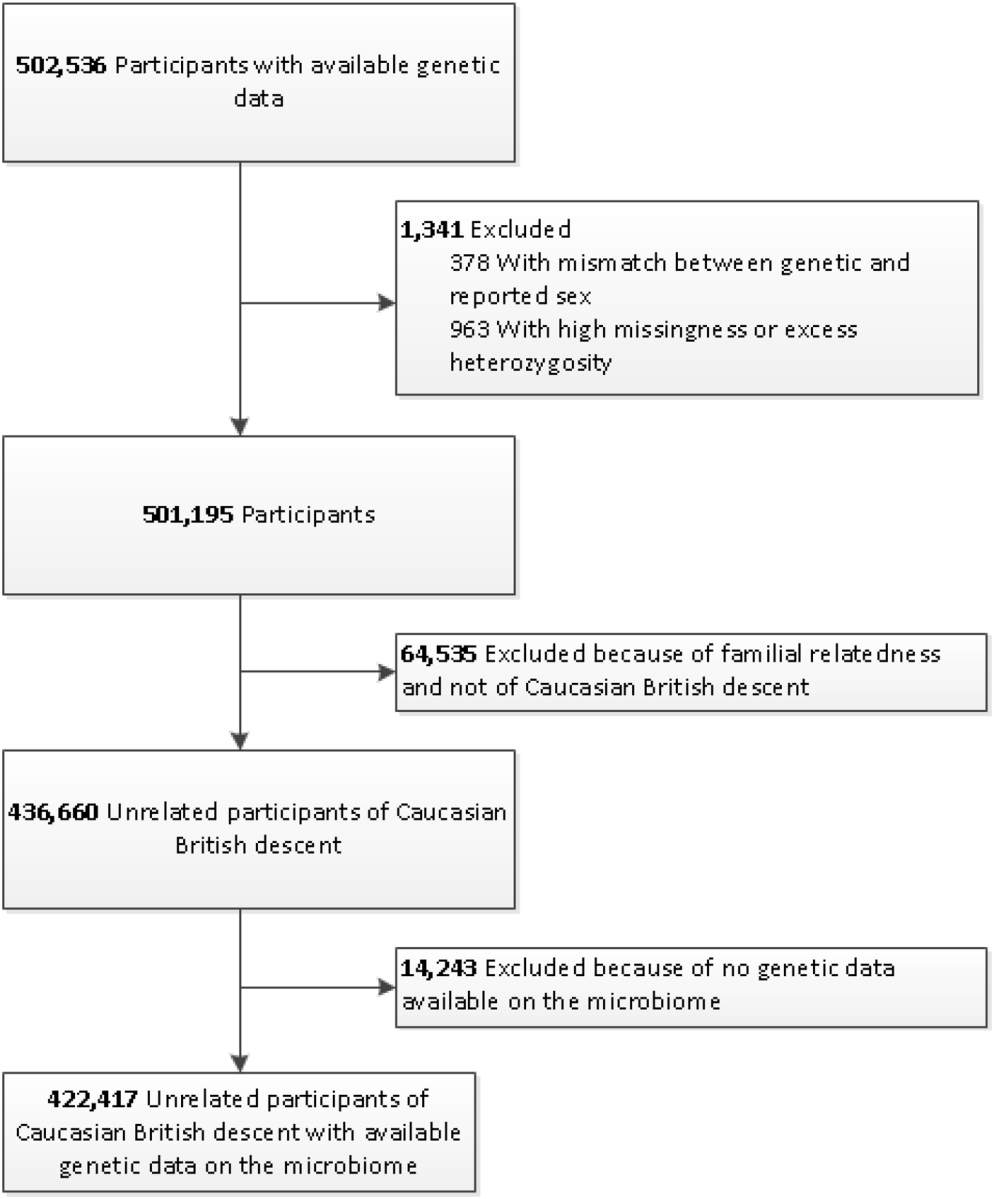
Flowchart for the selection of the analysed study sample from the UK Biobank Study.

We studied 422,417 unrelated individuals with a mean age of 57 ± 8 years, of which 194,567 (46%) were male (Table 1). Figure 1 presents a flowchart of the study sample selection. Median follow-up was 6 years (interquartile range (IQR) 5–7) and the median total exposure was 66-person years (interquartile range (IQR) 59–72).

**Table 1:** Baseline characteristics.

Characteristic	No
Total, no	422,417
Male	194,567 (46.1%)
Age, mean (SD), y	57 (8)
Years of exposure, median (IQR), y	66 (59–72)
Body mass index (kg/m^2^)	27.4 (4.8)
Systolic blood pressure (mmHg)	133 (20)
Diastolic blood pressure (mmHg)	82 (9)
Smoking behaviour	
Active daily	32,829 (8.0%)
Active occasionally	11,545 (2.8%)
Stopped ≤ 12 months	1,961 (0.5%)
Stopped > 12 months	131,800 (32.0%)
Never or < 100 cigarettes	233,186 (56.7%)
Medical history	
Hypertension	125,243 (29.6%)
Diabetes Mellitus type 2	16,815 (4.0%)
Hyperlipidemia	80,712 (19.1%)

*SD* standard deviation, *IQR* interquartile range.

### Phenome-wide scan on outcomes

**Figure 2:**
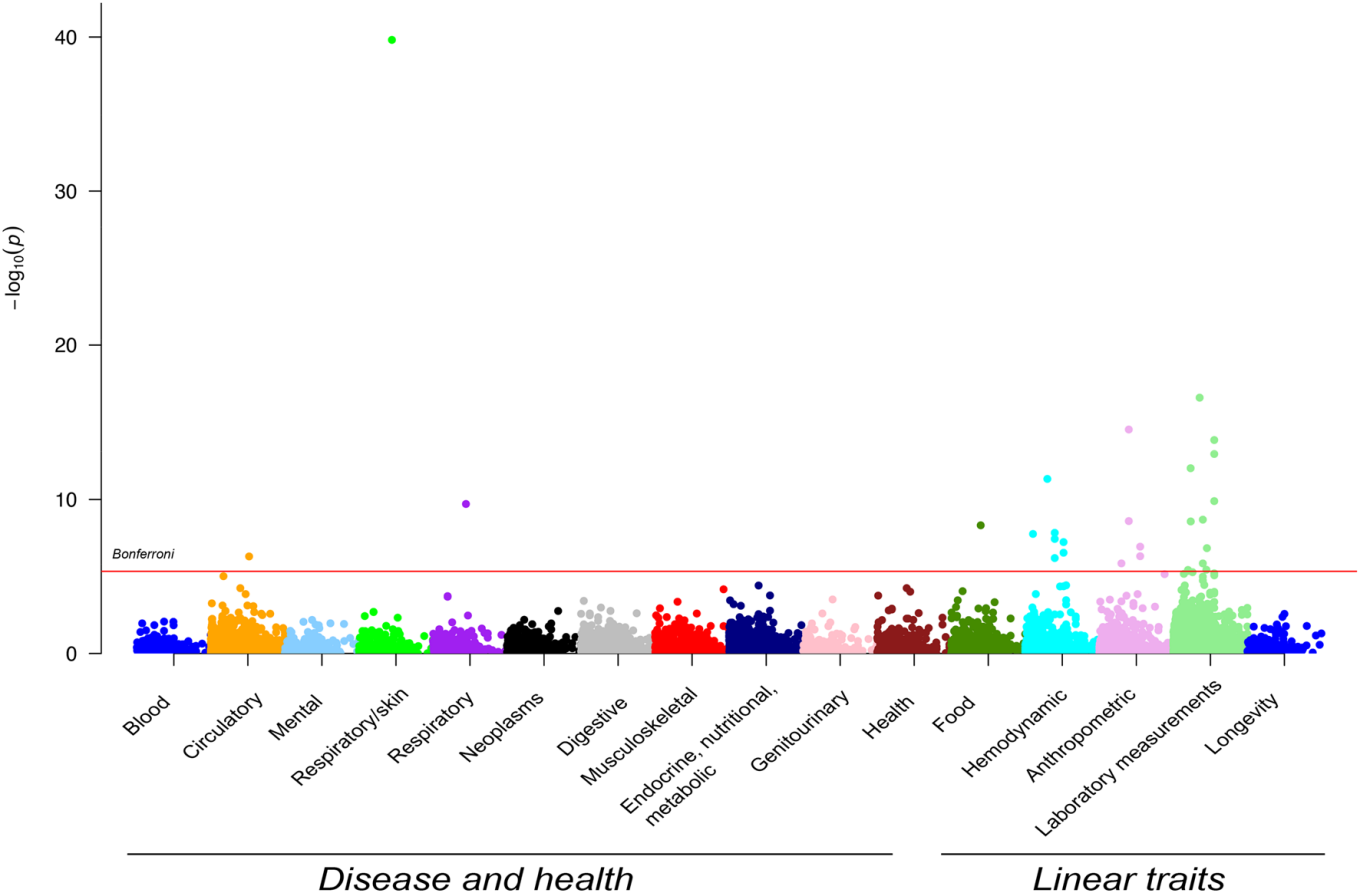
Manhattanplot for associations between microbiome SNPs and health and disease outcomes and continuous outcomes. On the x-axis, the health and disease outcomes (according to the ICD 10 code), and continuous are shown. On the y-axis, the *P*-value is shown. The red line indicates the significance threshold using Bonferroni correction.

A total of eleven microbiome SNPs was associated with 28 outcomes after a stringent Bonferroni correction (*P* value < 4.63·10^−6^) (Fig. 2). The strongest associations with binary traits were observed with diseases related to the following general ICD-10 categories: circulatory system, respiratory system, and respiratory/skin. Complementary information on associations between the SNPs, bacteria, and clinical outcomes is provided in Supplementary Table 1.

An even large number of significant associations with linear traits was discovered; especially health-related measurement including food intake, hemodynamic parameters, anthropometric and laboratory measurements were highlighted (Fig. 2). Extended information on the associations between the SNPs, bacteria, and continuous outcomes is provided in Supplementary Table 2. We identified no significant interactions between sex and genetically determined changes in microbiome.

**Table 2:** Studies used for SNP extraction.

Author (year)	Table	Cohort (N)	Age range	Method	Unit	N SNPs (before clumping)	Clumped (lowest taxa)	N SNPs (after clumping)	Note
Davenport et al.^51^	S7	Hutterites (n = 184)	18–75	16S rRNA	Abundance	1	No	–	
Bonder et al.^21^	S3	LifeLines deep (n = 984)	18–84	Metagenomics sequencing	Abundance	58	Yes	10	Allele frequencies obtained from HaploReg v3
Goodrich et al.^48^	S5	TwinsUK (n = 2,139)	18–89	16S rRNA	Relative abundance	25	No	NB	
Wang et al.^49^	T2	PopGen (n = 914), FoCus (n = 1,115)	25–83, 18–83	16S rRNA	Relative abundance	43	Yes	–	
Turpin et al.^50^	S6	GEM Project (n = 1,098)	6–35	16S rRNA	Relative abundance	58	Yes	56	Effect sizes obtained from log-normal model
Scepanovic et al.^52^	S13	Milieu Intérieur (n = 858)	20–69	16S rRNA	Relative abundance	188	Yes	48	

N SNPs denotes number of SNPs, S denotes Supplementary Table, T denotes Table. Please note that the studies from Rothschild et al. was not taken forward in the current analyses since effect sizes for discovered SNPs were not provided in the manuscript.

### Mendelian randomization analyses

#### Single SNP

**Figure 3:**
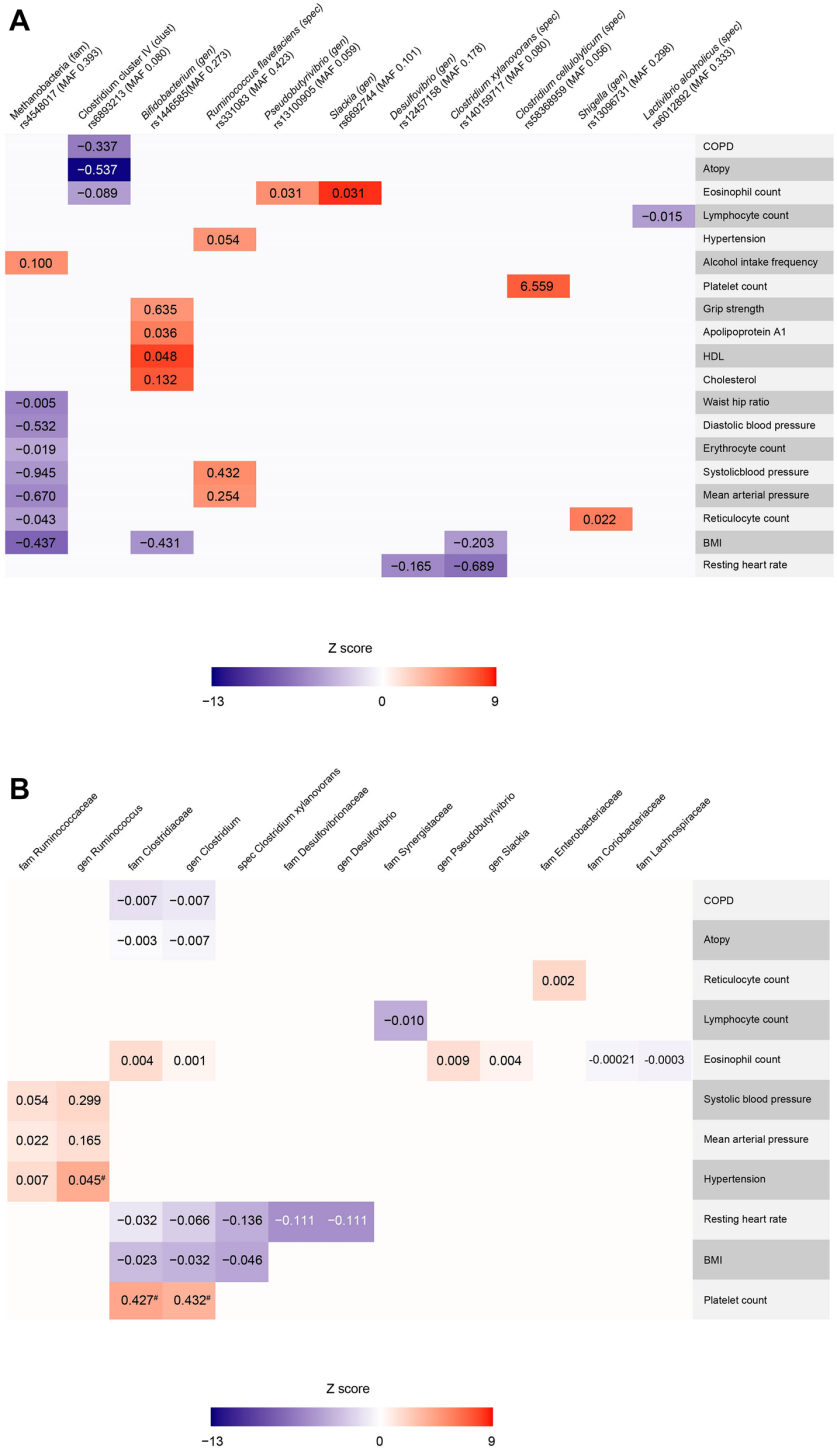
(**A**) Heatmap showing the Wald β’s of the associations between microbiome SNPs and health and disease outcomes, and continuous outcomes. Only significant associations (*P* value < 4.63·10^−6^) are shown. *BMI* body mass index, *COPD* chronic obstructive pulmonary disease, *HDL* high density lipoprotein, *MAF* minor allele frequency. (**B**) Heatmap showing the inverse variance weighted random effects β’s of the associations between the genetically determined increase of bacteria (family, genus and species level if possible) and health and disease outcomes, and continuous outcomes. Please note that the inverse variance weighted random effects β’s are in some cases liberal, considering the Rucker framework indicated unbalanced horizontal pleiotropy in the estimates as indicated by a significant (*P* < 0.05) Q–Q′ (and thus the MR-Egger estimate to be a better fit, please see Supplementary Table 5 for these results). *BMI* body mass index, *COPD* chronic obstructive pulmonary disease. ^#^ indicates suggestively significant.

Single SNP Mendelian randomization (MR) analyses using the Wald estimate were performed for all 28 outcomes. All Wald estimates of all 28 exposure-outcomes were significant (highest *P* value 4.4·10^−6^). Results are shown in Fig. 3A, are schematically depicted in Supplementary Figure 1 and full results can be found in Supplementary Table 3. F-statistics were all higher than 10, indicating little chance of weak-instrument bias based on the summary statistics. MR-steiger filtering indicated reversed causation to be likely in the association between *Clostridium cellulolyticum* and platelet count (*P* steiger < 5.6·10^−119^).

Under the ideal scenario that all MR assumptions are met, one could cautiously conclude the following based on these results. Genetically determined higher levels of Clostridium cluster IV were associated with a decreased risk for COPD (β − 0.34, SE 0.05, *P* = 1.96·10^−10^) and a decreased risk for atopy (β − 0.54, SE 0.04, *P* = 1.45·10^−37^). Genetically determined higher levels of the species *Ruminococcus flavefaciens* were associated with an increased risk for hypertension (β 0.05, SE 0.01, *P* = 4.97·10^−7^).

Genetically determined higher levels of the genera *Slackia* and *Pseudobutyrivibrio* were associated with an increase in eosinophil count. Higher levels of the species *Clostridium xylanovorans* were associated with a decrease in resting heart rate and body mass index (BMI). An increase of the genus *Desulfovibrio* was also associated with a decrease in resting heart rate. Genetically determined higher levels of Methanobacteria on family level were associated with an increase in alcohol intake frequency. Conversely, it was associated with a decrease in mean arterial blood pressure, systolic blood pressure, diastolic blood pressure, BMI, waist hip ratio, reticulocyte count, and erythrocyte count. Interestingly, genetically determined higher levels of the genus *Bifidobacterium* were associated with increased levels over cholesterol, apolipoprotein A1, HDL, and grip strength. This might seem paradoxical to previous findings in animals and human studies in which a favourable effect of specific *Bifidobacterium* strains on obesity and cholesterol levels was observed^26–28^.

A genetically increase in the genus *Shigella* was associated with an increase in reticulocyte count. Higher levels of Clostridium cluster IV were associated with a decrease in eosinophil count. Genetically determined higher levels of the species *Ruminococcus flavefaciens* were associated with an increase in systolic blood pressure and mean arterial pressure. Lastly, genetically determined higher levels of the species *Lactivibrio alcoholicus* were associated with a decrease in lymphocyte count.

#### Combined SNPs

To investigate whether the MR assumptions were truly met, we increased the amount of SNPs to assess the exposure-outcome association (Supplementary Table 3).

We were able to pool SNPs for a total of 26 exposure-outcome associations (Supplementary Table 4). Heterogeneity was indicated using Cochran’s Q statistic for all exposure-outcome associations (*P* < 0.05, Supplementary Table 5), except for the Clostridiaceae family and *Clostridium* genus with platelet count and the genus *Ruminococcus* with hypertension. This indicates at least balanced pleiotropy to be affecting the results. Given that assumptions of inverse variance weighted (IVW) effects model are violated under this scenario, we took forward the IVW random effects model as most liberal model (Fig. 3B, Supplementary Table 4). Compared to 27 causal estimates provided by the Wald ratio (one excluded by MR-Steiger filtering), now only 3 exposure-outcome associations remained suggestively significant [i.e. *P* < 0.05, but not significant for multiple testing (0.05/28)]. These were the causal estimates of the Clostridiaceae family and *Clostridium* genus with platelet count (β 0.43, SE 0.16, *P* = 7.28·10^−3^ and β 0.43, SE 0.19, *P* = 1.99·10^−2^; respectively) and the *genus* Ruminococcus with hypertension (β 0.45, SE 0.18, *P* = 1.11·10^−2^).

Intriguingly, the association between Clostridiaceae/*Clostridium* and platelet count remained significant considering the main driver (rs58368959, associated with *Clostridium cellulolyticum*) was removed as a result of MR-steiger filtering. The results were also robust to several sensitivity analyses, including MR-PRESSO and MR-Lasso, which are more outlier-robust, and the “unweighted” MR, which corrects for potential differences in effect size units as effect sizes depict relative abundance (Supplementary Table 4). The Rucker model showed no differences between the heterogeneity estimates of Cochran’s Q and Rucker’s Q, indicating at the absence of unbalanced horizontal pleiotropy and thus an inverse variance weighted model to be preferred (Supplementary Table 5). The results were not robust to MR-Egger, weighted median and weighted mode analyses (Supplementary Table 4). A forest and scatter plot of the MR between Clostridiaceae/*Clostridium* and platelet count can be found in Supplementary Figures 2–5.

The association between the genus *Ruminococcus* with hypertension was also robust to the “unweighted” MR, as expected considering the 2 SNPs included in the MR were obtained from the same study. Further sensitivity analyses could not be performed considering the amount of SNPs. One taxonomic level higher, 11 SNPs were pooled for the Ruminococcaceae family and hence more sensitivity analyses could be performed. The IVW random effect estimate was already insignificant (β = 6.94·10^−3^, SE = 6.63·10^−3^, *P* = 0.29). This suggests that, if the association between genus *Ruminococcus* and hypertension is true, it is specific for that genus within the Ruminococcaceae family. A forest and scatter plot of the MR between Ruminococcaceae/*Ruminococcus* and hypertension can be found in Supplementary Figures 6 and 7.

To investigate whether estimated effects of the Methano- and Bifidobacterium on associated outcomes were consistent when considering a community effect of the combined pool of gut bacteria, additional sensitivity analyses were performed (Supplementary Table 6). The results remained similar to the initial single SNP estimates.

Lastly, we performed a look-up in MR-Base, a platform for Mendelian randomization analysis, to explore whether the eleven SNPs were associated with other traits (*P*-value significance threshold of 5.00·10^−8^) in other studies than UK Biobank. The variant rs4548017 (Methanobacteria) was associated with ulcerative colitis, inflammatory bowel disease, and psoriasis^29,30^. The variant rs1446585 (*Bifidobacterium*) was associated with height^31^. We additionally performed a look-up in GeneCards for extra information about the genes related to the eleven significantly associated SNPs (Supplementary Table 7).

## Discussion

In the present study, we used a data-driven approach to identify human health and diseases parameters that are associated with genetic variants thought to influence the microbiome and identified 28 associations with 11 genetic variants. We found relevant associations with food intake, hypertension, BMI, lipids, atopy, and COPD. The possibility of a causal nature of these associations was tested using a MR approach. Although 27 out of 28 associations were indicated to be causal using a single SNP approach, only three of these associations were consistent across some, but not all sensitivity analyses.

Our findings highlight diseases that have been investigated in previous observational studies^32–41^. However, contrary to earlier observational research on the human gut microbiome and CVD, associations with CAD and MI did not reach statistical significance in our phenome-wide scan of microbiome associated SNPs^8,34^. While this might be due to a lack of study power or a small effect size of the evaluated genetic variants, associations with pathways via which the human gut microbiome may influence the development of CAD or MI (i.e. inflammation, arterial blood pressure, and circulating lipoproteins) were documented. As these factors are strongly related to CVD development, it is highly interesting to further unravel their, by microbiome influenced, effect in the long-term.

The association between a genetically determined increase of *Bifidobacterium* and elevated lipids is notable, since *Bifidobacterium* is considered to exert beneficial effects in human health^42^. Small sample sizes (n = 19–32 humans), differences in microbiome behaviour between obese and lean individuals, and the hypothesis that probiotic supplements may disturb the original composition of the microbiome could be possible explanations for this discrepancy^43–45^. In fact, a previous MR between genetically determined Bifidobacterium and HDL and LDL also found a positive causal estimate^46^. It is likely that total composition of the gut microbiome and bacteria ratios may eventually tell us more than single particular increases or decreases.

### Strengths and limitations

This study is the first to investigate the association between previously established microbiome SNPs and a wide variety of health and disease states simultaneously. The major strengths are the considerable sample size, the variety of explored outcomes without an a priori hypothesis, the prospective design of the UK Biobank study. This study could lead other researchers in certain directions to further explore the role of the gut microbiome in health and disease. In addition, we performed a large amount of MR sensitivity analyses (when enough SNPs were available). Another strength is that the MR was performed at different taxonomic levels. This allowed for the investigation of specific strains (species level) while also being able to increase the number of instruments when investigating at genus or family level. The potential importance was illustrated by the consistent estimates between Clostridiaceae family and *Clostridium* genus with platelet count, and the inconsistent estimates between the family Ruminococcaceae and genus Ruminococcus with hypertension. We believe this might provide insights how to perform and interpret MR estimates within the microbiome research.

We first address limitations most pertinent to a MR approach within microbiome research. It is of importance to note that the human gut microbiome is shaped for a large part by environmental factors^47^. However, the importance of a genetic component has been established as well^23,48^. In addition, the genetic approach allowed us to identify new pathways that are interesting to further unravel in the large cohort of the UK Biobank without real stool samples. A second limitation includes the lack of reliable SNPs associated with the gut microbiome. Although we adapted a fairly stringent *P* value threshold (5.00·10^−8^) for inclusion criteria and all F statistics were larger than 10, our results could still influenced by weak-instrument bias. “Winner’s curse”, i.e. overestimation of genetic associations within the dataset in which the SNPs were first identified, is likely considering the failure of replication of previously established SNPs^21,48–51^ in newer MWAS^47,52^. Since a two-sample approach was used without sample overlap, any bias due to weak instruments is directed to the null and therefore does not lead to false positive findings^53^. Another limitation is that the biological function through which the SNPs influence the gut microbiome is unsure and complex, especially considering the possible bilateral nature of the association within the GWAS exposure (i.e. the human microbiome influencing health status and health status influencing the human microbiome). We therefore performed an array of sensitivity analyses to assess whether the results of the single SNP estimates were robust to pleiotropic effects; this was not the case for most exposure-outcome associations. However, whether associations in multiple SNP MR analyses due to pleiotropic effects or weak instrument bias through the “winner’s curse” cannot be differentiated with the current set of analyses. In addition, we applied MR-Steiger filtering to evaluate potential reversed causation. We were unable to validate that the traits investigated share the same causal variant at a particular locus for both the exposure and outcome using colocalization methods, as we did not have the full Linkage Disequilibrium structure of the SNPs investigated. We did use MR-Base to check whether the SNPs were already known to be associated with other traits.

Considering the drawbacks of the current approach, we are very cautious in labelling the associations as “causal”. The current study should be considered as a broad hypothesis free scan in which causality of some associations is strengthened by contextualisation with previously described possible mechanisms (for example, the known risk factor hypertension in the development of CVD). In addition, it highlights the need for strict MR analyses in the microbiome context, as more extensively discussed previously^54^.

We believe that current study is of additive value to the multi-omics approach required to further dissect the role of the human microbiome in human health and disease^55^.

### Future perspectives

Our study could constitute a useful tool to identify bacteria of interest in order to thoroughly investigate the mechanisms between these bacteria and clinical outcomes; not only in the field of cardiology, but also in other fields. Second, the human gut microbiome might be used as a biomarker in disease risk stratification. Third, the observed associations can point towards new possibilities for therapeutic treatment. Furthermore, the abundance of particular bacteria or their metabolites could influence therapeutic treatment efficacy, as recently demonstrated in the treatment of Parkinson’s disease^56^.

## Conclusions

Human genetic determinants of the gut microbiome are associated with 28 specific health and disease outcomes including hypertension, atopy, COPD, lipids, and BMI. Multiple SNP MR pointed to a possible causal link between *Ruminococcus flavefaciens* and hypertension, and *Clostridium* and platelet count. Microbiota and their metabolites might be of importance in the interplay between overlapping pathophysiological processes, and could serve as potential therapeutic targets for the maintenance of health and prevention and treatment of diseases. However, many challenges remain in establishing causal relationships using current genetic data and approaches.

## Methods

### Study population

Data from the UK Biobank was accessed and analysed under an approved research proposal (application ID: 12006). The UK Biobank is a large community-based prospective study in the United Kingdom that recruited approximately 500,000 participants aged 40 to 69 years old with the general objective to improve the prevention, diagnosis, and treatment of diseases. All methods were performed in accordance with the relevant guidelines and regulations.The study design and population have been extensively described elsewhere^57^. All participants provided informed consent to participate^58^. The UK Biobank obtained approval from the relevant institutional review boards, that is, the North West Multi-centre Research Ethics Committee for the UK, the National Information Governance Board for Health and Social Care for England and Wales, and the Community Health Index Advisory Group for Scotland^59^.

### Identification of single nucleotide polymorphisms

Single nucleotide polymorphisms associated with the gut microbiome were collected from 6 previous GWASes^21,22,50–52,60^, using a genome-wide significance threshold of* P* < 5 × 10^−8^ for their inclusion in the current study. If the same SNPs and effect sizes were found across different taxonomic level within a single study, we took forward the association with the most differentiated taxonomic level to reduce multiple testing burden. SNPs were clumped within study per bacterium (on the lowest taxonomic level available) using the PLINK (version 1.9^61^) clumping procedure to prune genetic variants at a stringent linkage disequilibrium (LD) of *R*^2^ < 0.005 within a five megabase window. We did not clump the 25 SNPs from Goodrich et al*.* due to the character of the study (twin study instead of GWAS). In the end, this resulted in a total of 180 SNPs associated with the gut microbiome. For more information on the SNP selection, please see Table 2.

### Genotyping and imputation

The genotyping process and arrays used in the UK Biobank study have been previously described^59^. Briefly, participants were genotyped using the custom UK Biobank Lung Exome Variant Evaluation Axiom (Affymetrix; n = 49,949), which includes 807,411 SNPs, or the UK Biobank Axiom array (Affymetrix; n = 452,713), which includes 820,967 SNPs^59,62^. The arrays share over 95% of insertion and deletion markers^59,62^. Imputed genotype data were provided by the UK Biobank, based on merged UK10K and 1000 Genomes phase 3 panels^63^. Participants were excluded in case of missing genotype or sex mismatch (n = 378). Participants with high missingness or excess heterozygosity were also excluded (n = 963). Furthermore, 64,535 participants were excluded because of familial relatedness and being of non-Caucasian British descent. Lastly, participants without information available on the previously reported SNPs associated with the composition of the gut microbiome were also excluded (n = 14,243)^21,22^. Figure 1 presents a flowchart of the study sample selection.

### Definition of (cardiovascular) health, prevalent disease and new-onset disease

Two “health state” variables were generated. Cardiovascular health was defined as the absence of type 2 diabetes mellitus (DM2), stroke, and myocardial infarction (MI)^64^. Total health was based on the WHO top 10 causes of deaths in high-income countries. It was defined as the absence of coronary artery disease (CAD), stroke, Alzheimer’s disease, lung cancer, chronic obstructive pulmonary disorder (COPD), pneumonia, colon cancer, rectum cancer, DM, kidney diseases, and breast cancer at baseline and during follow-up^65^. The diseases was defined using self-reported diagnoses and medication (Hospital Episode Statistics data) as well as ICD-9 and ICD-10 codes as described previously^66^. Definitions of prevalent (established) and incident (new-onset) disease (outcomes) are shown in Supplementary Table 8.

### Vital signs, blood count, and food intake

Vital signs and biological samples were collected during the baseline visit together with data of self-completed questionnaires (including food questionnaires), interviews, and physical measurements. Blood pressure was measured twice for consistency and the average value was used. Automated measurements were corrected according to previously described methodology^67^.

### Phenome-wide scan on outcomes

Outcomes with a prevalence of ≤ 1% were excluded from the analyses. Logistic regression modelling was performed to assess the effect of microbiome SNPs on combined prevalent and new-onset diseases, and health outcomes in term of the resulting odds ratios (OR) and corresponding 95% confidence intervals (CIs). Genotypes were coded according to a dose–response model (0, 1, 2), where the allele coded with a 2 corresponded with an increased abundance of a certain bacterium. Linear regression analyses were conducted to assess the effect of microbiome SNPs on continuous outcome measures (anthropometric, hemodynamic and laboratory variables as well as longevity and food intake at baseline) in terms of the resulting β coefficients and associated standard errors (SE). Since sex differences exist regarding the human gut microbiome, we tested for sex-interactions in the association between the SNPs and all outcomes. All regression analyses were adjusted for age (at the moment of the last follow-up or age at baseline visit), sex (except when sex-interactions were tested), genotyping chip, and the first 30 principal components (to adjust for population structure) provided by the UK Biobank. A stringent Bonferroni correction was used to correct for multiple testing (180 SNPs, 60 outcomes; 0.05/10,800 = *P* value < 4.63·10^−6^). Outcomes reaching statistical significance after Bonferroni correction were considered of interest for further analyses (see Mendelian randomization section below). Analyses described above are performed using Stata version 15 (StataCorp, Texas, United States).

### Mendelian randomization analyses

In order to explore the possibility of a causal relationship between the exposure (the gut microbiome) and the outcomes reaching statistical significance after Bonferroni correction, we first performed a single SNP Mendelian randomization (MR) using the Wald-estimate^68^. Weak instrument bias was assessed per SNP using the F-statistic, calculated with the following formula: F = R^2^(*n* − 2)/(1 − R^2^). In this formula, *n* is the sample size of the exposure and R^2^ is the amount of variance of the exposure explained by the SNP^69^. R^2^ was calculated using a previously established formula^70^. Allele frequencies and SNP position were obtained for the SNPs obtained from the study of Wang et al.^71^*.* In order to explore the possibility of reversed causation to be a driver of the current results, we applied MR-Steiger filtering. MR-Steiger filtering calculates the R^2^ for the exposure and outcome and removes variants if the R^2^ of the exposure is significantly lower (*P* value < 0.05) than the R^2^ of the outcome^72^. R^2^ for linear traits was calculated as mentioned above^70^. R^2^ for binary outcomes was calculated on the liability scale according to previously established methods^73^. These results can be found in Supplementary Table 3.

Pleiotropy within MR analyses refers to a genetic variant having multiple effects and distorts MR estimates when genetic variants affect the outcome independently. Given the uncertainty of the biological mechanisms underlying the SNP-exposure association, and hence uncertainty on whether the genetic variants affect the outcome independently, we aimed to gain additional insight in pleiotropy by increasing the amount of SNPs to assess the exposure-outcome association. SNPs associated with the same exposure and their effect estimates on the outcome (possibly not reaching Bonferroni significance) were obtained from the pool of all 180 SNPs. This resulted in an additional 153 exposure-outcome associations, for which single SNP MR estimates, F-statistics^69^ and MR-Steiger^72^ results can be found in Supplementary Table 3 as well. By definition, effect sizes used to obtain pooled estimates should reflect the same unit. We therefore only took forward SNPs with effect sizes reflecting relative abundance, given the fact that (a) only one SNP (rs4548017) reached statistical significance on the tested outcomes after Bonferroni correction, (b) 169 out of 180 SNPs reflected relative abundance and c) the relative effects better reflect the biology of the *milieu intérieur*. Due to difference in in-depth classification of the taxonomic rank in the SNP-exposure association between and within study, SNPs were pooled on species, genus and family level. Again, SNPs were clumped (between-studies) for every bacterium per level. Detailed information on the amount of SNPs per bacterium per taxonomic level and the outcomes tested can be found in Supplementary Table 4. Three methods were applied based on the amount of genetic variants available: (a) if after pooling only one SNP remained available, only Wald ratio, MR-Steiger filtering^72^ and F-statistics^69^ were calculated as explained above (28 exposure-outcome associations, see Supplementary Table 3), (b) if two SNPs were available, inverse variance weighted fixed effects and random effects were calculated; *I*^2^- index^74^ and Cochran’s Q^75^were used to assess heterogeneity and differentiate between models (6 exposure-outcome associations) (Supplementary Tables 4 and 5) and (c) if more than two SNPs were available, a more extensive pallet of analyses was performed (22 exposure-outcome associations) (Supplementary Tables 4 and 5). First of all, this extended pallet included the Rucker framework. Heterogeneity was and thus potential pleiotropy was first assessed in the IVW effect estimate using Cochran’s Q statistic. A *P* value of < 0.05 was considered as an indication of at least balanced horizontal pleiotropy, indicating the IVW random effects model to be preferred over a fixed effects model. heterogeneity and, as a consequence, of pleiotropy. Next, the MR-Egger test was performed^76^. The MR-Egger relies on different assumptions than the IVW method, as it does not assume all instruments to be valid by allowing for a non-zero intercept. The intercept therefore represents unbalanced horizontal pleiotropy. The MR-Egger intercept, the standard error and p-value were therefore calculated. A MR-Egger intercept *P* value of < 0.05 was considered to be an indicator of unbalanced horizontal pleiotropy. Heterogeneity was assessed within the MR-Egger by calculating Rucker’s Q. A significant difference between these two heterogeneity statistics (Q–Q′, *P* < 0.05) was considered indicative of unbalanced horizontal pleiotropy as well and thus the MR-Egger test to be the preferred method over the IVW. *I*^2^_GX_ was calculated to assess weak-instrument bias within MR-Egger estimates^77^. An *I*^2^_GX_ of >> 95% was considered to indicate a low risk of weak-instrument bias within the MR-Egger test. In addition, MR-Lasso^78^ and MR-PRESSO^79^ (the latter when 4 or more SNPs were available) were performed, two tests that are robust to outliers due horizontal pleiotropy when the SNPs used are valid instrumental variables. Lastly, weighted median^80^ and weighed mode^81^ effect estimates were calculated. These methods allow for estimation of causal effect estimates when the majority (> 50%) or the plurality (no larger subset of invalid instruments estimating the same causal parameter) of SNPs are valid instruments.

Under scenario (b) and (c), we additionally performed an “unweighted” MR by calculated an inverse variance weighted fixed and random effects estimate after equally weighting all genetic instruments (i.e. negative and positive beta’s were set to − 1 and 1 respectively) and were assumed to be estimated with infinite precision (i.e. the stand error was set to 0). This was performed considering that relative abundance might still reflect different biological implications between studies/populations. We recalculated Cochran’s Q using these fixed weights to differentiate between the fixed and random effects model.

MR analyses were performed using R (version 3.6.3), the TwoSampleMR package 0.5.3^82^, MR-PRESSO^79^ (version 1.0), and the source code for MR-Lasso^78^ in a Two-Sample MR setting as proposed in the article of Rees et al.

### Conditional analyses on the methano- and bifidobacterium

To investigate whether estimated effects of the Methano- and Bifidobacterium on associated outcomes were consistent when considering a community effect of the combined pool of gut bacteria, we performed additional sensitivity analyses. First, we took forward SNPs containing (a) taxonomic information up to family level and (b) effect sizes depicting relative abundance, totalling to 136 SNP. After clumping on family level, 32 unweighted GRS for 32 different bacterial families were constructed. These families included the Acidaminococcaceae, Bacteroidaceae, Barnesiellaceae, Bifidobacteriaceae, Clostridiaceae, Coriobacteriaceae, Desulfovibrionaceae, Enterobacteriaceae, Erysipelotrichaceae, Eubacteriaceae, Firmicutes, Lachnospiraceae, Lactobacillaceae, Leuconostocaeae, Marinilabiliaceae, Micrococcaceae, Mogibacteriaceae, Moraxellaceae, Odoribacteraceae, Paenibacillaceae, Pasteurellaceae, Peptococcaceae, Peptoniphilaceae, Porphyromonadaceae, Rhodospirillaceae, Rikenellaceae, Ruminococcaceae, Streptococcaceae, Synergistaceae, Veillonellaceae, Victivallaceae and the Bifidobacteriaceae/Methanobacteriaceae family (for the association of the Methanobacteriaceae/Bifidobacteriaceae respectively). We opted for an unweighted GRS based on the same rationale as the choice for performing an “unweighted” MR as sensitivity analysis. These were subsequently used as covariates in the SNP (rs4548017 and rs1446585 for the Methano- and Bifidobacteriaceae, respectively)-outcome association in the UK Biobank.

### Look-ups

We performed a look-up in MR-Base, a platform for Mendelian randomization analysis, to explore whether the SNPs would be associated with other traits in order to gain insights in potential pleiotropic effects^82^. Furthermore, we consulted GeneCards for extra information about the genes related to the analysed SNPs^83^.

## Supplementary information

Supplementary Information 1.

Supplementary Information 2.

## Data Availability

Data is available on request.
