# Supplementary material for: Human genetic determinants of the gut microbiome and their associations with health and disease: a phenome-wide association study
Source: Sci Rep. 2020 Sep 8;10:14771. doi: 10.1038/s41598-020-70724-5 (PMC7479141; doi:10.1038/s41598-020-70724-5)
Supplement: Supplementary file 1 — Supplementary Information 1. [file 41598_2020_70724_MOESM1_ESM.docx]

**Human genetic determinants of the gut microbiome and their associations with health and disease**

**Supplementary Figures**

Hilde E. Groot, M.D.^1^; Yordi J. van de Vegte, B.Sc.^1^; Niek Verweij, Ph.D.^1^; Erik Lipsic, MD., Ph.D.^1^; Jacco C. Karper, M.D., Ph.D.^1^; Pim van der Harst, M.D., Ph.D^1,2^.

1. Department of Cardiology, University Medical Center Groningen, University of Groningen, Groningen, The Netherlands

2. Department of Cardiology, Division Heart & Lungs, University Medical Center Utrecht, University of Utrecht, the Netherlands

**
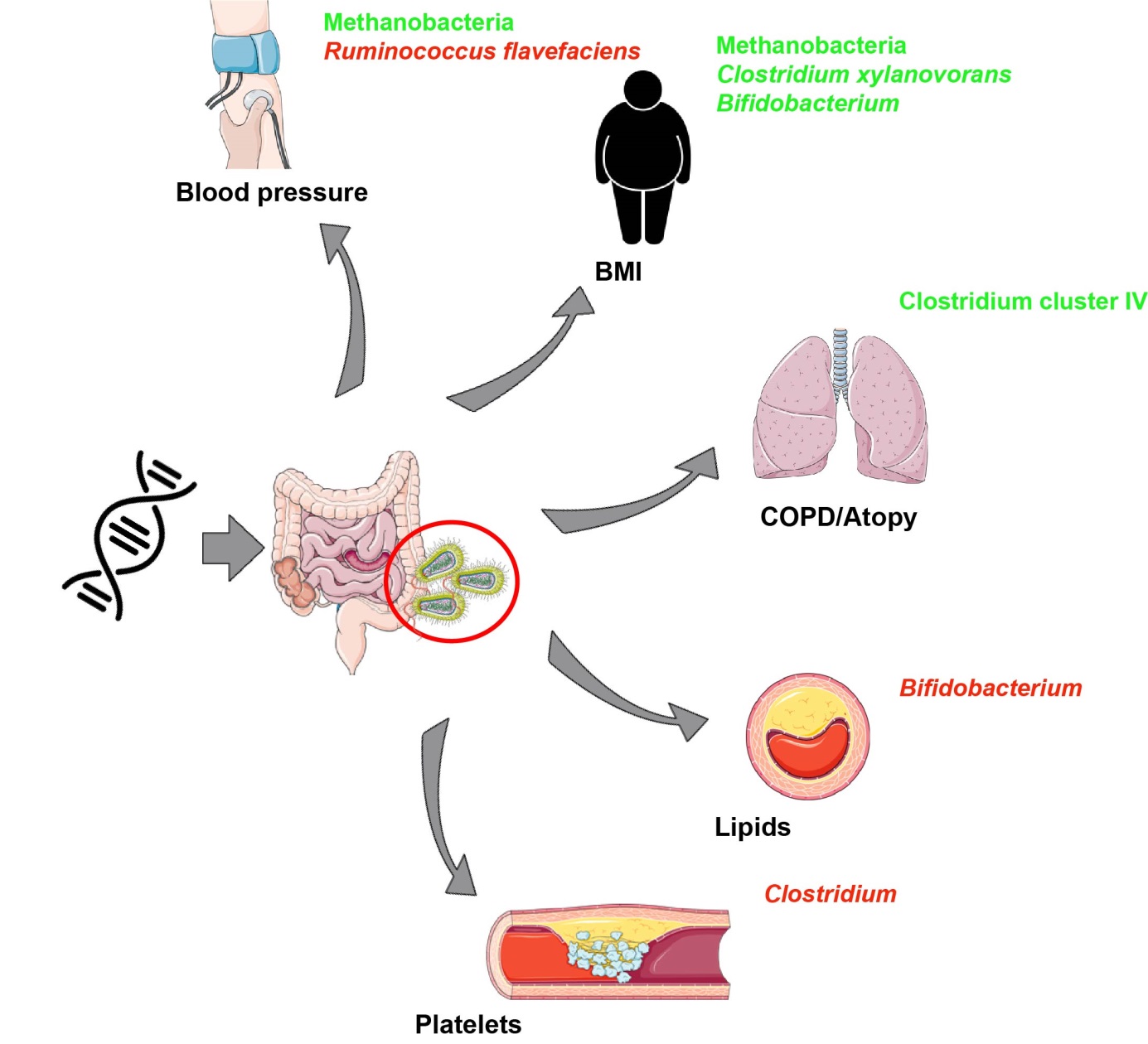
**

Supplementary Figure 1. Overview figure of the potential associations found between SNPs shown to be associated with the human gut microbiome and disease outcomes in the UK Biobank. Created with use of images from Smart Servier Medical Art (<https://smart.servier.com/>). Adobe Illustrator CC 2017 was used.


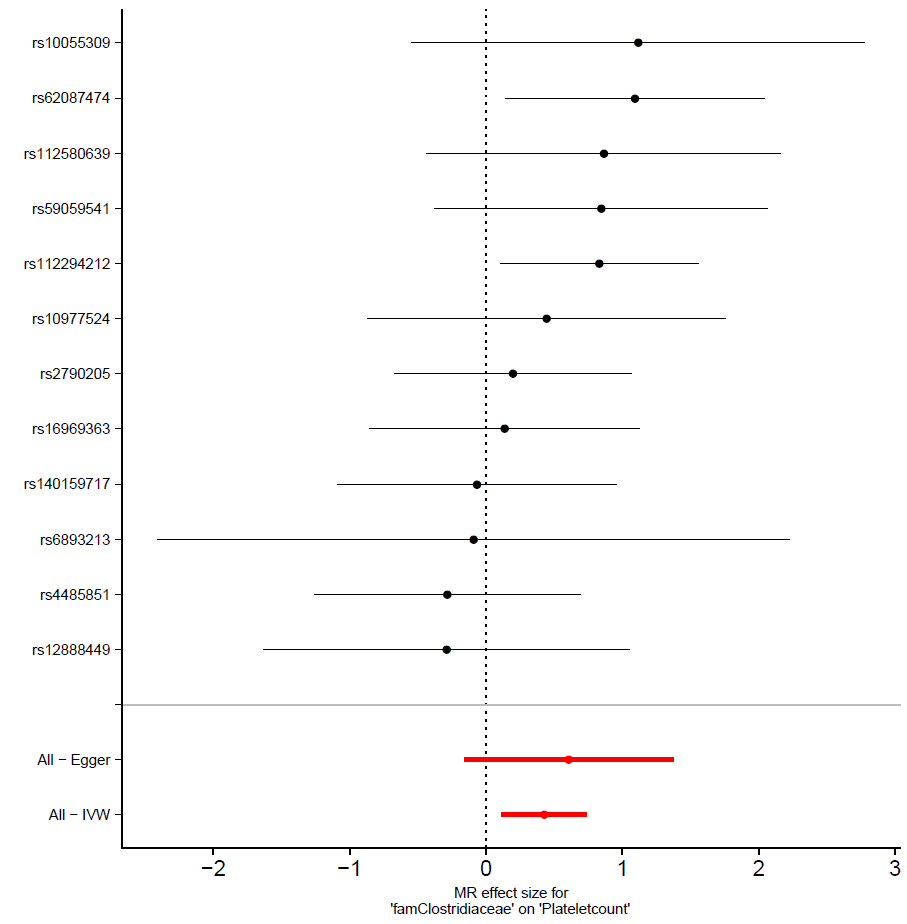


Supplementary Figure 2. The Mendelian Randomization effect size and standard error of exposure on outcome product are displayed on the X-axis. The different genetic variants for exposure are listed on the Y-axis.


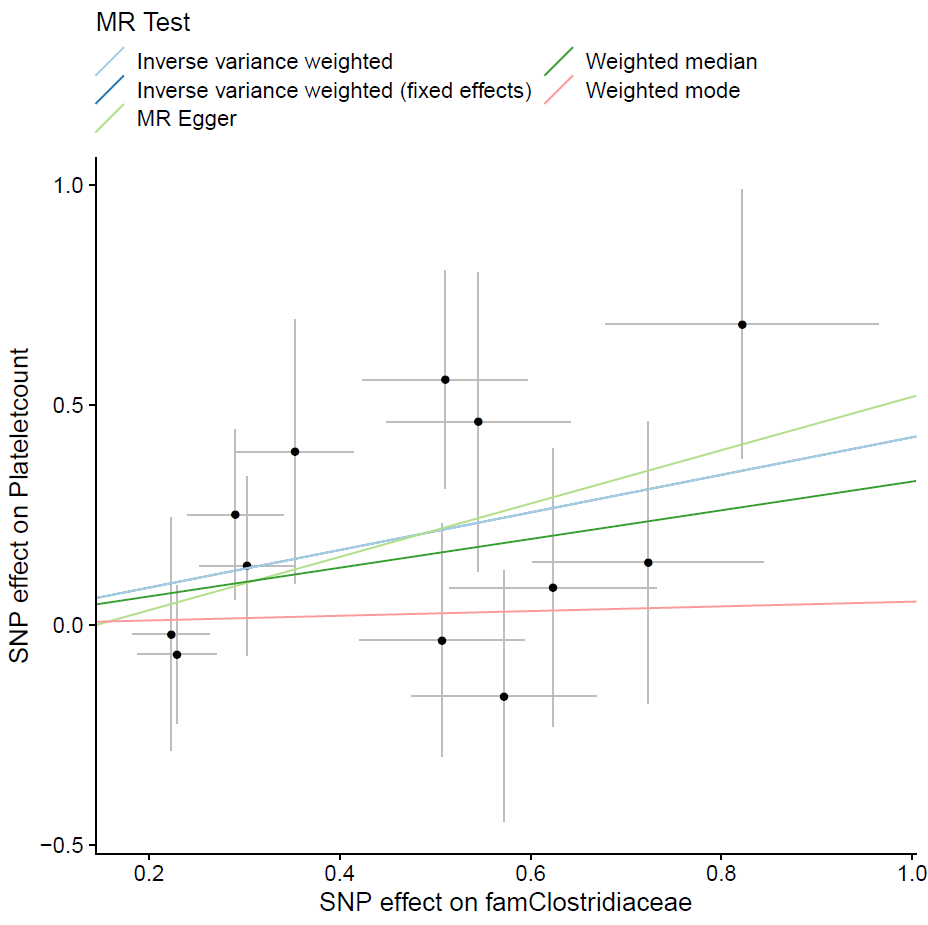


Supplementary Figure 3. Scatter plot including the MR estimates between exposure and outcome, in which a threshold of *P* < 1·10^−8^ was used for the selection of variants. The variants’ effect size and standard error on exposure are displayed on the X-axis, the variants’ effect size and standard error on outcome on the Y-axis.


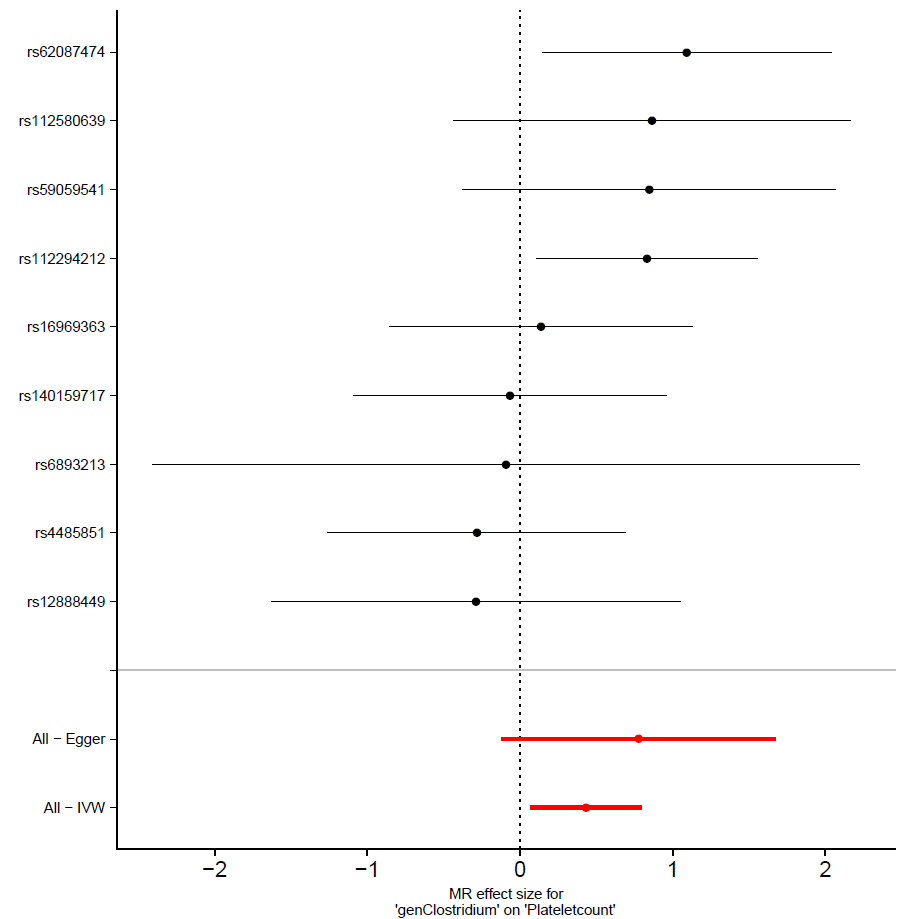


Supplementary Figure 4. The Mendelian Randomization effect size and standard error of exposure on outcome product are displayed on the X-axis. The different genetic variants for exposure are listed on the Y-axis.


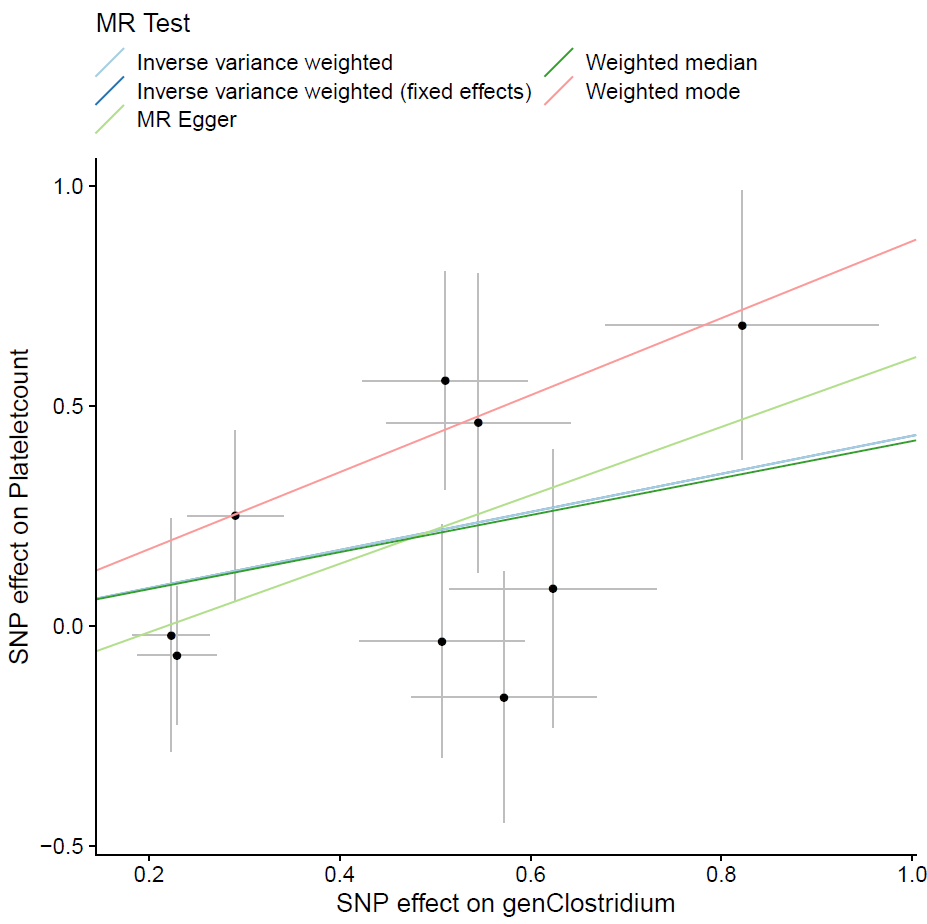


Supplementary Figure 5. Scatter plot including the MR estimates between exposure and outcome, in which a threshold of *P* < 1·10^−8^ was used for the selection of variants. The variants’ effect size and standard error on exposure are displayed on the X-axis, the variants’ effect size and standard error on outcome on the Y-axis.


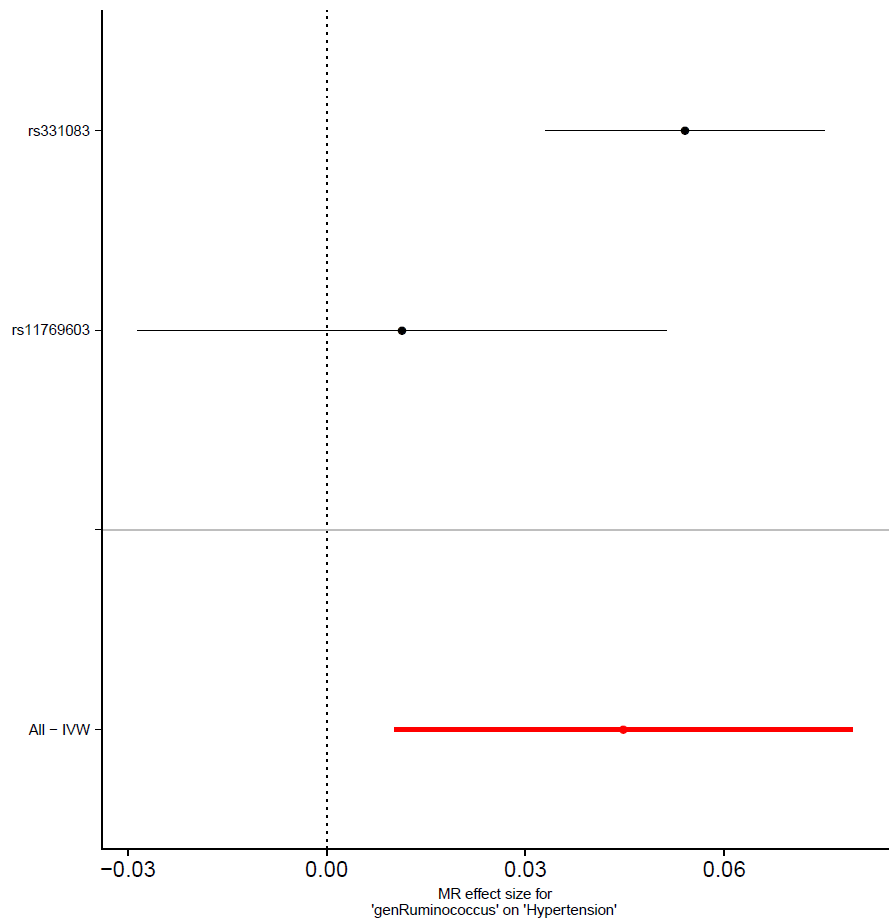


Supplementary Figure 6. The Mendelian Randomization effect size and standard error of exposure on outcome product are displayed on the X-axis. The different genetic variants for exposure are listed on the Y-axis.


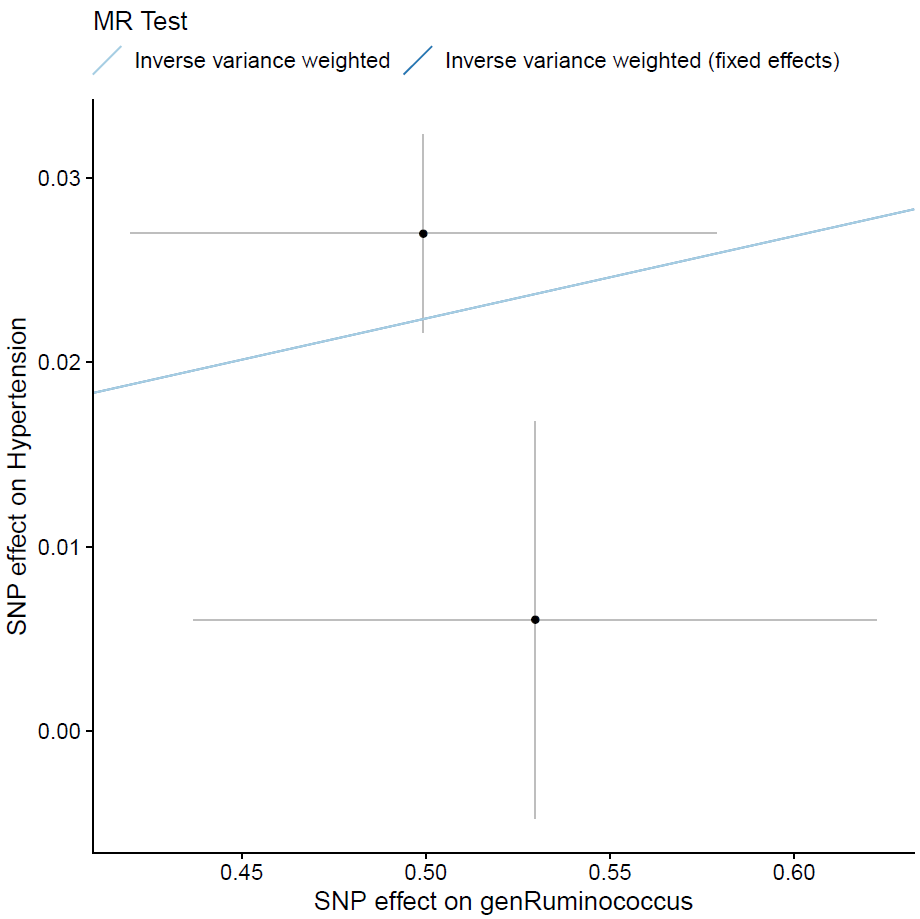


Supplementary Figure 7. Scatter plot including the MR estimates between exposure and outcome, in which a threshold of *P* < 1·10^−8^ was used for the selection of variants. The variants’ effect size and standard error on exposure are displayed on the X-axis, the variants’ effect size and standard error on outcome on the Y-axis.
